# QuPath Algorithm Accurately Identifies MLH1-Deficient Inflammatory Bowel Disease-Associated Colorectal Cancers in a Tissue Microarray

**DOI:** 10.3390/diagnostics13111890

**Published:** 2023-05-28

**Authors:** Ross J. Porter, Shahida Din, Peter Bankhead, Anca Oniscu, Mark J. Arends

**Affiliations:** 1 Edinburgh Pathology, CRUK Scotland Centre, Institute of Genetics and Cancer (IGC), University of Edinburgh, Scotland EH4 2XU, UK; ross.porter@ed.ac.uk (R.J.P.);; 2 Edinburgh IBD Unit, Western General Hospital, NHS Lothian, Scotland EH4 2XU, UK; sdin@exseed.ed.ac.uk; 3 Edinburgh Pathology, CRUK Scotland Centre, Centre for Genomic & Experimental Medicine, Institute of Genetics & Cancer, University of Edinburgh, Scotland EH4 2XU, UK

**Keywords:** QuPath, machine learning, biomarker, MLH1, colorectal cancer, inflammatory bowel disease, mismatch repair, immunohistochemistry, histology

## Abstract

Current methods for analysing immunohistochemistry are labour-intensive and often confounded by inter-observer variability. Analysis is time consuming when identifying small clinically important cohorts within larger samples. This study trained QuPath, an open-source image analysis program, to accurately identify MLH1-deficient inflammatory bowel disease-associated colorectal cancers (IBD-CRC) from a tissue microarray containing normal colon and IBD-CRC. The tissue microarray (*n* = 162 cores) was immunostained for MLH1, digitalised, and imported into QuPath. A small sample (*n* = 14) was used to train QuPath to detect positive versus no MLH1 and tissue histology (normal epithelium, tumour, immune infiltrates, stroma). This algorithm was applied to the tissue microarray and correctly identified tissue histology and MLH1 expression in the majority of valid cases (73/99, 73.74%), incorrectly identified MLH1 status in one case (1.01%), and flagged 25/99 (25.25%) cases for manual review. Qualitative review found five reasons for flagged cores: small quantity of tissue, diverse/atypical morphology, excessive inflammatory/immune infiltrations, normal mucosa, or weak/patchy immunostaining. Of classified cores (*n* = 74), QuPath was 100% (95% CI 80.49, 100) sensitive and 98.25% (95% CI 90.61, 99.96) specific for identifying MLH1-deficient IBD-CRC; κ = 0.963 (95% CI 0.890, 1.036) (*p* < 0.001). This process could be efficiently automated in diagnostic laboratories to examine all colonic tissue and tumours for MLH1 expression.

## 1. Introduction

Accurate histological assessment including immunohistochemistry is necessary to confirm a diagnosis of cancer and guide treatment decisions. Tissue biomarker studies also depend on the accurate interpretation of immunostains. Previous studies have demonstrated high inter-observer variability when identifying tissue histology or assessing immunostain patterns and intensities [1,2,3]. For example, a multicentre study reported poor inter-observer agreement for HER2 immunostain interpretation in breast cancer, with kappa 0.2–0.6; absolute agreement for immunohistochemistry was found for only one out of three cases [4]. The variability in the interpretation of tissue type and staining can be minimised by expert pathologists and independent validation of the immunohistochemistry, respectively [5,6,7]. However, this process is still limited by human factors such as the subjective evaluation of immunostain intensity or perceived proportion of stained cells [8]. Other human factors can impact immunostain interpretation and thus increase inter-observer variability. For example, Butter and colleagues recently demonstrated that a pathologist’s personality can impact the interpretation of PD-L1 immunostaining in non-small cell lung cancer specimens [9].

It is often important to identify rare or uncommon phenomena within a larger patient cohort (e.g., to identify an infrequently mutated protein by immunohistochemistry within a biomarker discovery cohort). This is becoming especially important in the era of personalised medicine where specific mutations or combinations of mutations may suggest efficacy of specific targeted therapies. Identifying rare staining patterns amongst thousands of tissue samples is labour-intensive and time consuming, even when tissue microarrays (TMAs) are used. With integration of clinical data, digital image analysis platforms may also identify important relationships between biomarker expression and clinical outcomes [10].

Artificial intelligence (AI)-assisted digital image analysis previously represented an expensive futuristic ideal for biomarker- and histopathology-based research. Application was limited by expensive and cumbersome scanning equipment, poor capacity for storing high-quality whole slide images, underdeveloped image processing software, and a lack of freely available, user-friendly AI software. These barriers have since been overcome and there are now several user-friendly software packages for digital pathology analysis, including the open-source software QuPath [11].

QuPath is a free, open-source, digital image analysis platform designed to allow users to view and interrogate whole slide images [11]. The software has many tumour identification and high-throughput biomarker evaluation tools available. It allows batch-processing and has scripting functionality. QuPath also allows users to create and share software extensions to solve new clinically relevant problems [11]. It has an easy-to-use interface, meaning that computer programming skills are not required, which improves clinical translational ability. Many other image analysis tools exist, such as ImageJ [12], Fiji [13], Icy [14] and CellProfiler [15]. Each software has its own benefits and disadvantages—for example, ImageJ may be superior in some aspects of image analysis capability; however, it is unable to work smoothly with large whole slide images. There are no comparative studies which reflect the different focus of each image analysis platform.

Colorectal cancer (CRC) is one of the most common malignancies worldwide, representing one in ten cancer cases and deaths [16]. Despite bowel cancer screening programmes and therapeutic advances, the 5-year survival for colorectal cancer is around 60% [17]. Most cases of CRC are described as sporadic: they develop through the adenoma-carcinoma pathway, whereby normal colonic mucosal crypt epithelium neoplastically transforms into adenomatous polyps with dysplasia, and a proportion of these may evolve into invasive adenocarcinoma [18]. The majority of sporadic CRCs are characterised by early and frequent *APC* mutations and late and moderately frequent *TP53* mutations [19]. Other CRC phenotypes exist and are clinically important. For example, patients with inflammatory bowel disease (IBD) have an increased risk of developing CRC compared to the general population [20,21]. The most common IBD phenotypes are ulcerative colitis and Crohn’s disease. The incidence of CRC may be >60% higher in patients with IBD compared to the general population (95% CI for Crohn’s disease is 20–200%; 95% CI for ulcerative colitis is 30–200%) [22]. Patients with IBD develop more aggressive cancers that often present late and in younger patients [23,24,25,26]. The pathophysiology of IBD-CRC is different to sporadic CRC and is characterised by early *TP53* mutations and late and infrequent *APC* mutations; whereas a minority of around 13–15% sporadic CRC develop defective DNA mismatch repair as a result of acquired promoter hypermethylation of the *MLH1* gene that silences its expression, and these occur mostly in the right colon [27,28]. Patients who develop colorectal cancer on a background of inflammatory bowel disease (IBD-CRC) have higher recurrence rates and much poorer survival rates (two-fold increase in mortality) compared with patients who develop sporadic CRC, which makes this an important cohort to study [24,25]. We have recently comprehensively reviewed IBD-CRC [29].

Within healthy colorectal mucosal crypt epithelium, the DNA mismatch repair (MMR) pathway is essential for repairing DNA replication-associated errors that include both base mismatches (such as A pairing with C or G, rather than with the usual T nucleotide) and repetitive sequence errors, often termed microsatellites (such as CACACACA becoming CACACACACA following DNA slippage during replication) [30]. Inactivating mutations or absence of MMR proteins in tumour cells can result in defective mismatch repair (dMMR), which increases the mutation rate due to unrepaired DNA replication errors within the tumour cells, elevating the likelihood of acquiring further cancer gene mutations [30]. MMR-deficient colorectal cancers represent approximately 13–15% of sporadic colorectal cancers resulting from *MLH1* promoter hypermethylation, and in addition, there is a further 3% CRC due to Lynch syndrome, a genetic tumour predisposition syndrome conferred by inheritance of DNA variants affecting any one of the four MMR genes *MLH1*, *MSH2*, *MSH6* and *PMS2* [31,32,33,34,35]. We have previously reported that deficient MMR due to loss of MLH1 protein expression occurs in >25% of all inflammatory bowel disease-associated colorectal cancers [36]. MMR-deficient IBD-CRC are an important cohort to study as these patients have tumours with high mutation rates generating a high neo-epitope load indicating that they may respond well to immunotherapy [31,36]. Manually identifying MLH1-deficient tumours from immunostained biopsies is time consuming and labour-intensive.

Therefore, the aim of this study is to determine whether it is possible to train QuPath to accurately identify MLH1-deficient IBD-CRCs from a TMA containing both normal colon and IBD-CRC samples.

## 2. Materials and Methods

### 2.1. Tissue Microarray and Immunohistochemistry

A previously characterised MLH1 immunostained TMA was used in this study [36]. In brief, 34 patients with IBD-CRC or normal colonic mucosa were identified (Supplementary Table S1). H&E-stained sections from formalin fixed paraffin embedded biopsies were reviewed by an expert pathologist to determine suitable areas for macro-dissection; 0.6 mm cores were used to create the TMA (*n* = 147 cores + 15 ‘blank’ cores for slide special orientation). Immunohistochemistry for MLH1 was undertaken as previously described [36], and histology and staining patterns were independently assessed by an expert pathologist, blind to all data.

### 2.2. Patient Clinico-Pathological Characteristics

Characteristics are summarised in Supplementary Table S1. IBD-CRC cases consisted of 15 (44.1%) females and 19 (55.9%) males. Sixteen (47.1%) patients had a diagnosis of Crohn’s disease and 18 (52.9%) patients had a diagnosis of ulcerative colitis. Median age at cancer diagnosis was 65 (Q1 51, Q3 73.25) years. Eight cancers were in the caecum, 1 cancer was at the ileocaecal valve, 5 cancers were in the ascending colon, 1 cancer was at the hepatic flexure, 3 cancers were in the transverse colon, 1 cancer was in the right colon without an exact location specified, 1 cancer was at the splenic flexure, 1 cancer was in the descending colon, 2 cancers were in the sigmoid colon, 3 cancers were at the recto-sigmoid junction, 7 cancers were in the rectum, and 1 cancer was in the ano-rectum. Thirty-two (94.1%) cancers were adenocarcinomas, 9 (28.1%) of which had mucinous differentiation and 1 (3.1%) had signet ring cell morphology. Two (5.9%) cancers were squamous cell carcinomas arising either in the anorectum or in the caecum. At resection, 11 (32.4%) patients had evidence of metastasis to at least local lymph nodes.

### 2.3. Digital Image Analysis

The MLH1-stained TMA slide was scanned using the NanoZoomer scanner (Hamamatsu Photonics (UK) Ltd., Welwyn Garden City, UK) to generate a high-resolution brightfield whole slide image. This scanner is routinely used in many laboratories to provide crisp high-quality whole slide images [37]. The open-source quantitative pathology and bioimage analysis program QuPath (v0.2.1) was used for analysis [11]. QuPath software was chosen for this study as it allows digital analysis of whole slide images and tissue microarrays with creation of novel algorithms for machine learning. These capabilities are not available in most other open-source software programs. Our group has also previously used this software successfully [10,11].

The slide was uploaded into the QuPath software as a Brightfield (H-DAB) image. Initially, a new training image was created manually by selecting 14 representative regions of tissue, with a measured size of 500 μm × 500 μm. These 14 regions represented diverse tissue types and staining patterns. Using this training image, colour deconvolution was performed using the ‘estimating stain vectors’ command to optimise haematoxylin (0.67253, 0.56452, 0.47856), 3,3′-diaminobenzidine (DAB) (0.25141, 0.41193, 0.87585), and background (235, 232, 239) detections. To detect MLH1-positive cells, Positive Cell Detection was performed using a single-intensity threshold parameter of 0.2 (see Table 1 below). Smooth object features were added with radius (FWHM) 25 μm and restricted to objects with the same base classification. To detect tissue histology, the wand and brush tools were then used to annotate regions of tissue to classify ‘normal epithelium’ versus ‘tumour’ versus ‘immune cell infiltrates’ versus ‘stroma’. ‘Immune cell infiltrates’ were defined as a collection of immune cells, including lymphoid aggregates and lymphoid follicles, and were classified as separate from ‘stroma’ as their morphology is different—these separate classifications were to ensure the QuPath algorithm did not mistakenly identify lymphoid aggregates for epithelial cells or tumour cells. The object classifier was trained based on all prior detections using a random trees classifier, with all feature and class measurements included. ‘Live update’ was used to provide real-time feedback to allow focused training of the software. Once complete, the classifier was saved and the training image closed.

Attention then turned back to the initial image uploaded into QuPath. Using this digitally scanned whole-slide TMA image, the TMA dearrayer tool was used to identify individual cores from A1 to I18: size was set at 0.9 mm for each TMA core, there was an identification density threshold which was set at 5.0, and there was a bounds scale factor which was set at 105.0. Dearraying accuracy was visually assessed for every core, and the TMA grid was adjusted manually as and where required. Most unsuitable cores were correctly marked as ‘invalid’ by QuPath; however, a small number of further unsuitable cores were manually marked as ‘invalid’ where appropriate.

The protocol that had been optimised on the training image—to detect both MLH1 protein expression and tissue histology type (i.e., ‘normal epithelium’ versus ‘tumour’ versus ‘immune cell infiltrates’ versus ‘stroma’)—was then applied to the TMA. This consisted of colour deconvolution → positive cell detection → add smooth features → load object classifier.

**Figure 1:**
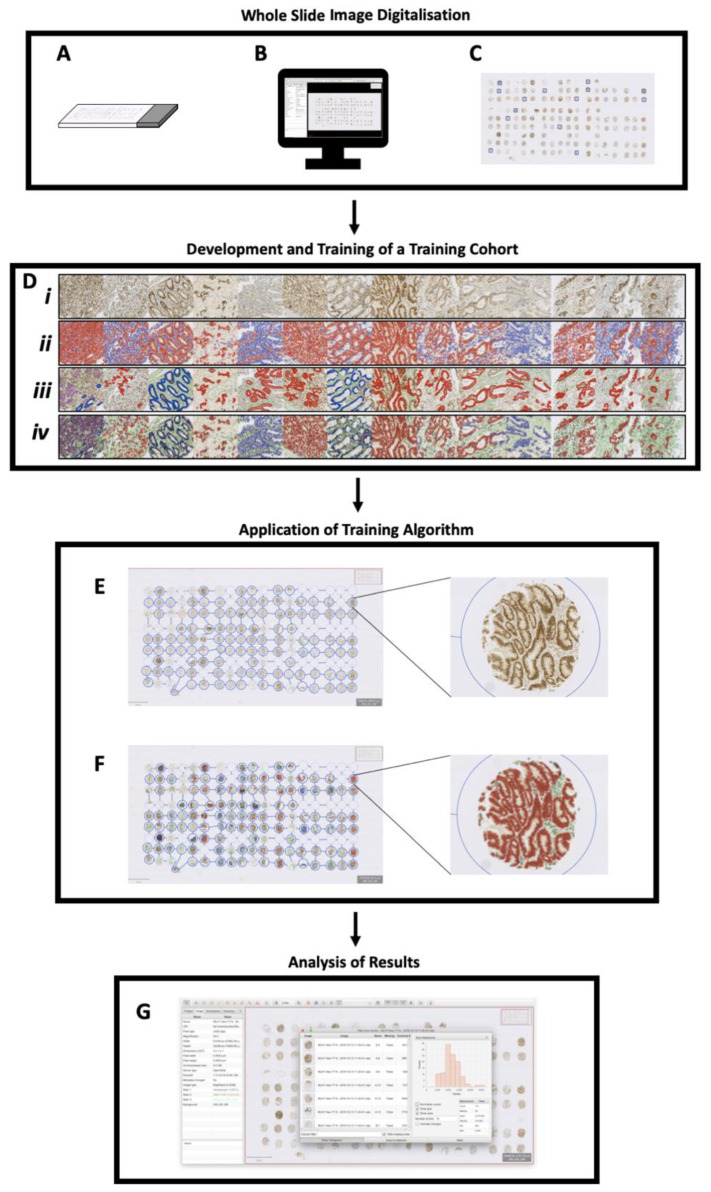
Protocol for identifying MLH1-deficient IBD-CRC tumours: (**A**) tissue microarray is immunostained for MLH1 (protein of interest); (**B**) slides are digitalised and whole slide images imported into QuPath; (**C**) small representative regions (*n* = 14; each 500 μm × 500 μm) are used to create a training image; (**D**) (***i***) The training image is created and colour deconvolution optimised; (***ii***) positive cell detection is run and optimised, with smooth features added; (***iii***) wand and brush annotation tools define various tissue types and train the object classifier; (***iv***) live update function allows real-time feedback to improve the object classifier, which is saved; (**E**) using the original digitalised slide, the TMA dearrayer tool is applied; (**F**) the optimised training algorithm settings, outlined in (**D**) (***i***–***iv***) are applied along with the saved object classifier; (**G**) the measurement table is viewed and exported for statistical analysis.

**Figure 2:**
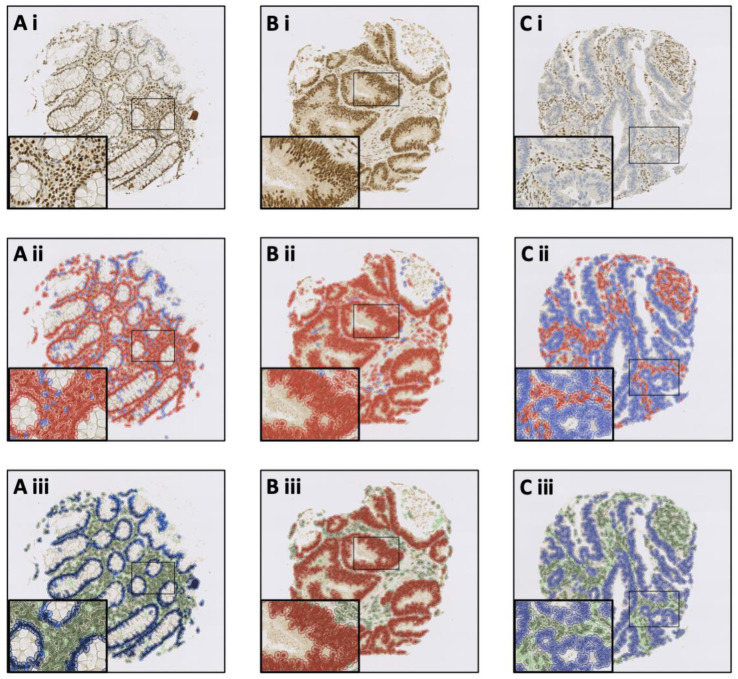
QuPath software trained to recognise MLH1 status and tissue histology. Photomicrographs demonstrate QuPath’s potential to be trained to identify: (**A**) MLH1-proficient normal colonic epithelium; (**B**) MLH1-proficient IBD-CRC; and (**C**) MLH1-deficient IBD-CRC. Rows represent: (**i**) digitalised MLH1 immunostained TMA cores; (**ii**) positive cell detection where red represents MLH1-positive cells and blue represents MLH1-negative cells; and (**iii**) final cell classification where dark blue represents MLH1-positive normal epithelium, sky blue represents MLH1-negative normal epithelium, red represents MLH1-positive IBD-CRC, light blue represents MLH1-negative IBD-CRC, dark green represents MLH1-positive stroma, and light green represents MLH1-negative stroma. Dark (MLH1-positive) and light (MLH1-negative) purple represent immune cell infiltrates and are not seen in these examples.

TMA measurements were exported for statistical analysis. A methodological summary (Figure 1) and detection/object classification examples (Figure 2) are illustrated below with cell detection and object classification photomicrographs shown in Figure 2. 

### 2.4. Statistical Analysis

**Figure 3:**
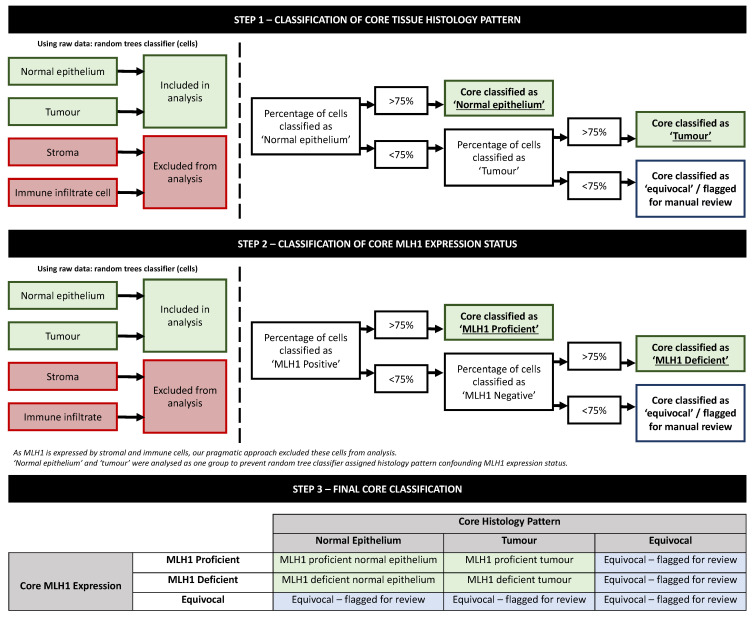
Decision tree flowchart illustrating steps to identify core histology and MLH1 status following random trees classification.

QuPath output data were exported to Microsoft Excel (V16.22) and analysed to identify the percentage of ‘normal epithelium’ and ‘tumour’ cells per core. Data were also analysed to identify the percentage of MLH1-positive and MLH1-negative cells (within cells classified as either ‘normal epithelium’ or ‘tumour’ histology patterns only; stroma and immune cell infiltrates data were disregarded). Thereafter, histological diagnosis and MLH1 status were confirmed/certified if ≥75% of cells were designated as either normal epithelium or tumour, or MLH1-positive or MLH1-negative, respectively. MLH1 status and tissue histology pattern (normal epithelium versus tumour) were assessed separately to prevent confounding. For example, if a tissue core contained ≥75% normal epithelium and ≥75% of cells (normal epithelium or tumour cells) were identified as proficient for MLH1, the core would be classified as ‘MLH1 proficient normal epithelium’. Cores were flagged for manual review if the 75% threshold that had been agreed prior to data analysis was not met. The explanatory decision tree flowchart, informed by the random tree classifier, is outlined in Figure 3. Data were then coded and exported to IBM^®^ SPSS^®^ (V25.0). Diagnostic agreement was assessed between our algorithm’s classification and expert pathologist assessment (blind to algorithm data) using sensitivity and specificity analysis with associated Cohen’s kappa (κ) tests. A two-tailed alpha was set at 0.05, and 95% confidence intervals (CI) are supplied where appropriate. 

### 2.5. Qualitative Analysis

All tissue cores that the QuPath algorithm flagged for manual review were reviewed by 3 observers (MJA, RJP, SD). Cores were reviewed independently by each observer and then discussed as a group to reach consensual agreement—through discussion +/− re-reviewing cores. A statement was written pertaining to why the core had been flagged for manual review by QuPath (Supplementary Table S2). This was agreed upon by all observers. One observer (RJP) reviewed these statements to identify key ‘themes’. These ‘themes’ were also agreed upon by all 3 observers.

## 3. Results

### 3.1. QuPath Identifies TMA Cores Valid for Assessment

First, we wanted to determine whether QuPath could identify cores and tissue that were suitable for assessment. QuPath identified 139/162 (85.8%) cores as valid for assessment and 23/162 (14.2%) cores as invalid for assessment through the TMA dearrayer function, based upon estimated tissue area. No core coded as invalid by QuPath was thought valid on manual review of each core. On manual review, 26 further cores were thought to be invalid, mostly due to lack of histologically diagnostic epithelium (e.g., cores with predominant connective tissue). Of valid cores, the TMA dearrayer template position only had to be adjusted slightly for three (2.65%) cores due to folded or fragmented tissue (i.e., atypical core appearance).

### 3.2. The Trained QuPath Algorithm Accurately Identifies MLH1 Status and Core Histology

Next, we wanted to determine whether our algorithm, which was trained from a small training dataset of 14 representative tissue regions (each 500 μm × 500 μm), was able to identify correct overall core histology (i.e., tumour versus normal epithelium) and MLH1 status (i.e., absent versus present) within the TMA. Accuracy was assessed against independent expert histopathologist review of each core, blind to QuPath data.

Specifically, the QuPath algorithm was able to identify both the correct overall core histology and MLH1 status in (73/99, 73.74%) cases. Incorrect identification was reported in 1/99 (1.01%) case.

The algorithm identified 25/99 (25.25%) cores for manual review. Cores were flagged for review due to uncertain overall core histology (16/99, 16.16%), uncertain overall core MLH1 status (8/99, 8.08%), or both (1/9, 1.01%). Cores flagged for review were excluded from sensitivity and specificity analysis and underwent manual review for thematic analysis.

### 3.3. The Trained QuPath Algorithm Is Sensitive and Specific for Identifying Core Histology and MLH1 Status

Our algorithm had very high sensitivity (100% (95% CI 80.49, 100)) and specificity (98.25% (95% CI 90.61, 99.96)) for identifying MLH1-deficient IBD-CRC from all other cores in the TMA (i.e., MLH1-deficient IBD-CRC versus MLH1-proficient normal epithelium or MLH1-proficient IBD-CRC—there were no cores of MLH1-deficient normal epithelium, as expected). Diagnostic agreement between QuPath and the expert GI pathologist was therefore very high with κ = 0.963 (95% CI 0.890, 1.036) (*p* < 0.001).

### 3.4. Five Major Categories Were Identified as the Reason for the Trained QuPath Algorithm Flagging Cores for Review

There were 25 cores that the algorithm flagged for manual review. Sixteen cores were flagged for histology, eight cores were flagged for MLH1 expression status, and one core was flagged for both histology and MLH1 expression status. Manual qualitative review of these 25 flagged cores revealed that the reasons for this fell into five categories: (1) small quantity of tissue, (2) atypical tumour morphology, (3) excessive inflammatory/immune infiltration, (4) normal mucosal crypts (no tumour), or (5) weak or patchy immunostain intensity. Often, a combination of these reasons was observed. Qualitative assessment of each flagged core is reported in Supplementary Data.

## 4. Discussion

Current methods for expensively trained human histopathologist-driven accurate identification of specific tissue histopathological diagnoses and their associated immunohistochemical staining patterns are labour-intensive, time consuming, and often confounded by inter-observer variability [1,2,3]. Digital image analysis using artificial intelligence (AI) algorithms, such as QuPath, has lately emerged as a beneficial tool [11]. The ability to use AI to screen large numbers of samples for both diagnosis of tumour versus not tumour as well as for immunostaining positively or negatively, based on tissue type, histological features, and protein expression status, has high research and clinical utility. However, there are no published studies describing or validating this approach. In this study, we demonstrate that QuPath can be trained to accurately identify both tissue histopathological diagnostic patterns and MLH1 protein expression status with very high sensitivity and specificity within a TMA.

The original QuPath paper describes the software’s ability to work effectively with TMA whole slide images—locating, identifying, and analysing tissue cores [11]. Previous studies have also successfully used QuPath to identify positive and negative immunostained cells, for example, after immunostaining for Ki-67, mutated proteins, and immune cells [10,38,39]. Some studies have also trained QuPath algorithms to differentiate between tissue compartments [40,41]. Our study contributes to validating digital image analysis, via QuPath, as an important tool in the era of AI-based machine learning and digital image analysis.

A recent study in this field by Reichling and colleagues demonstrates that AI software could classify tissue structure, tumour cell characteristics, and immune cell infiltrates, and this can be extrapolated to predict CRC outcomes [42]. The authors initially used QuPath for image analysis; however, their approach to analysis was much more complex, using a superpixel strategy (a complex pixel classification approach) and developing AI software. Indeed, using a pixel classifier may feed more information into an AI algorithm; however, this was suboptimal for our study where the simpler object classifier worked superiorly. Analysis in our study was not run or recorded after initially attempting to use a pixel-based strategy, due to inferior performance. There are several possible reasons for this, such as our samples being arranged in a TMA, which means there was only a small and focused amount of tissue available for analysis. In summary, the simpler object classifier method worked effectively with a TMA, which allowed straight-forward, fast, efficient, and accurate analysis. Pixel classification strategies could be better suited to larger tissue samples, and this warrants further investigation. Nonetheless, it is important to select a methodological approach that suits both the tissue available and questions to be answered.

A novel strength of our study was our approach to interpreting the raw image analysis data in a way that can tolerate errors in cell identification and classification, which are inevitable when applied to real-world datasets. To this end, our algorithm flagged around a quarter of the TMA cores for manual review, and the main reason for this was due to uncertainty about core histology pattern. To our knowledge, this capability has not been previously demonstrated. Figure 2 represents an ideal; in reality, our random trees classifier misidentified ‘normal epithelium’ and ‘tumour’ cells within some cores (Supplementary Data), which reduced QuPath’s ability to firmly diagnose every core. Reasons for uncertainty about tissue histology pattern included using a large and diverse IBD-CRC cohort with some atypical tumour morphologies (including poorly differentiated adenocarcinomas with reduced cohesion, and adenocarcinomas with mucinous differentiation or signet ring carcinoma cell morphology), having only a small quantity of tissue per core with lack of supporting stromal tissue architecture, an excessive inflammatory/ immune cell infiltrate, and including normal epithelium in the TMA as normal colorectal epithelium had small nuclei with less distinct morphology. Reasons for uncertainty about MLH1 expression status were more straightforward to explain: the immunostain was sometimes patchy and thus suboptimal for most of these problematic cores. For MLH1 immunohistochemistry, differing staining intensities could simply represent ineffective epitope retrieval or variation in epitope fixation (known to be a common problem for resected colorectal cancers undergoing variable fixation), rather than a biologically significant phenomenon [43]. This is likely to confer interpretative challenges for colorectal cancer cores in which lymphocytes or stromal cells do not strongly express MLH1 on immunohistochemistry. Therefore, it is important that these sections have been flagged for manual review by expert pathologists. Alternatively, weak/patchy MLH1 immunostaining intensity, when there is strong lymphocytic or stromal cell MLH1 expression, may represent protein destabilisation as a genuine abnormality of mismatch protein expression and therefore represent an important subset of IBD-CRCs. While applying intensity thresholds could be an effective approach to subcategorising these samples, it would also risk incorrectly classifying cases with poorly fixed tissue [43]. As recently discussed by Campanella and colleagues [44], as we would assume the pathologist operates with 100% sensitivity and specificity, it is not the goal for an AI algorithm to outperform the expert. Instead, it is important to maintain very high sensitivity with an acceptable specificity and a reasonable manual review rate as shown here. In our study, the manual review rate may be reduced further if our cohort excluded normal colonic epithelium (i.e., only contained IBD-CRC samples). This is clinically feasible as histological diagnosis is made by an expert pathologist for every biopsy/resection sample. 

**Figure 4:**
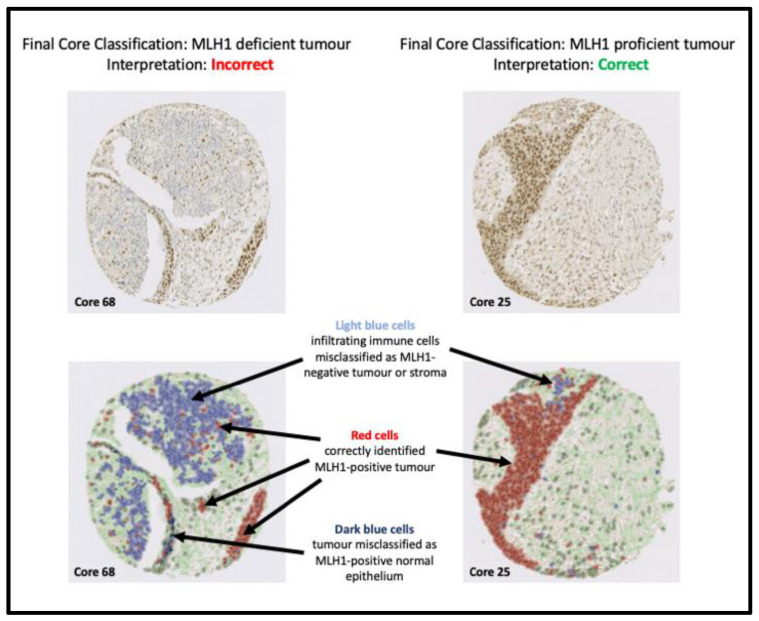
Our algorithm misclassified Core 68 as an MLH1-deficient tumour when it is an MLH1-proficient tumour. This was because the random trees classifier misclassified a large MLH1-deficient ‘immune cell infiltrate’ as ‘tumour’, which confounded MLH1 assessment. Most immune cells were MLH1-negative; however, there are occasional MLH1-positive immune cells, which could represent proliferating lymphocytes. A small number of stromal cells were also misclassified. Immune cell infiltrates and stromal cells are ignored for MLH1 assessment to eliminate confounding issues; however, it was incorrectly included in this instance as immune cells had been misclassified as tumour cells. Core 25 was taken from the same patient as Core 68 and correctly classified by our algorithm, due to more accurate recognition of MLH1-positive tumour cells by the random trees classifier as illustrated in the annotated images.

The AI-based algorithm misclassified 1/99 (1.01%) core as MLH1-deficient tumour when the diagnosis was MLH1-proficient tumour. This was because the random trees classifier did not recognise atypical tumour morphology and misclassified a large MLH1-deficient ‘immune cell infiltrate’ as ‘tumour’, which confounded MLH1 assessment. Immune cell infiltrates could be disregarded for tissue MLH1 assessment to eliminate confounding; however, it was included in this instance as immune cells had been incorrectly classified as tumour cells. The TMA included two other cores from the same patient: in this case, one of these cores was correctly classified as MLH1-proficient tumour and the other core was flagged for manual review. This is illustrated in Figure 4.

Therefore, to address misclassification issues, further training of the QuPath algorithm may be needed to recognise atypical tumours which are very heavily infiltrated by immune cells. Further, future algorithms could consider analysing two biopsies from every patient and ‘flag for review’ any incongruous classifications. However, it is reassuring that the two other cores from the aforementioned same patient were not misclassified, and our overall misclassification rate was very small.

A limitation of this study was that we used a TMA. This meant that many cores had suboptimal quality or were missing, and were thus removed from assessment. While TMAs are widely used in research studies and clinical trials, they are not used in routine diagnostics. Therefore, it is important for future work to validate this methodology on whole tissue biopsies or resected tumour block sections immunostained for MLH1, as these samples are currently used for diagnostic assessment and reporting in pathology laboratories. Indeed, whole tumour slides have more complex tissue architecture and are more representative of diagnostic routines. As clinical laboratories have standardised tissue processing and immunohistochemistry protocols, it is unlikely that a new algorithm would need to be trained for each individual sample. In addition, the use of biopsies or whole tumour slides may help to reduce the number of specimens flagged for manual review when compared with TMA cores. For example, with regards to thematic analysis (Section 3.4), there would be a larger quantity of diagnostic tissue available for QuPath assessment, and immune infiltration or lymphoid aggregates or lymphoid follicles would thus represent a smaller proportion of the slide. However, this requires validation in the clinical setting using many samples from different laboratories. To expand clinical application, future work should also assess whether this methodology—training a new algorithm—could be applied to identify other target proteins in other tissue types with similar sensitivity, specificity, and manual review rates.

It is important for histopathologists to be able to determine the mismatch repair status (proficient versus deficient) in both colorectal cancers and endometrial cancers, the two major cancers seen in Lynch Syndrome, for the purpose of screening for Lynch Syndrome, as recommended by national guidelines [45,46]. This approach may also be applicable to the much longer list of tumours at other organ sites, to which Lynch Syndrome also confers increased susceptibility. In addition, 13–15% of sporadic colorectal cancers and 25–30% of sporadic endometrial cancers are defective for mismatch repair as a direct result of bi-allelic promoter hypermethylation of the *MLH1* gene, and this is relevant as it alters the prognosis of these tumours and patients with metastatic dMMR cancers may become eligible for immunotherapy using highly effective immune check point blockade agents such as anti-PD-1 (Pembrolizumab) or anti-CTLA4 (Nivolumab) monoclonal antibodies [31,32,45,46]. Previously, we have shown that in excess of 25% of IBD-CRCs are defective for mismatch repair, mostly as a result of *MLH1* gene promoter hypermethylation, with a small number of mutations affecting any one of the four MMR genes, and these cancers, when metastatic, may also be considered for immune checkpoint inhibition based on the results of MMR immunohistochemistry [36], as shown here. 

## 5. Conclusions

In this proof-of-concept pilot study, an MLH1-immunostained TMA of cores of inflammatory bowel disease-associated colorectal cancers, we demonstrate that QuPath can be trained using a small tissue cohort to identify tissue histopathological diagnostic patterns and MLH1 expression status with very high sensitivity and specificity. Therefore, this streamlined methodology, utilising QuPath, can be used to simply and efficiently identify a small but clinically important cohort of patients with MLH1-deficient IBD-CRC. This is clinically relevant as MLH1-deficient IBD-CRCs have a high neo-epitope load and thus may respond well to immunotherapy, such as immune check point blockade [36]. Therefore, our AI-based algorithm could potentially be used to screen all tissue samples stained with MLH1 in pathology departments to identify this important cohort of patients. If integrated effectively, positively identified specimens could be flagged for histopathologist attention early, ultimately reducing time from biopsy to commencement of therapies. Further, accurate manual assessment of immunostains could reduce the need for labour-intensive evaluation in both clinical and research settings. This proof-of-concept methodology could also be applied to other clinically relevant immunostains and pathological abnormalities. For example, it could be used to detect other important cancer biomarkers in different tissue types, such as PDL-1 positive non-small cell lung cancers or B-RAF positive melanomas. Large validation studies are now eagerly awaited.

## Figures and Tables

**Table 1:** Optimised positive cell detection settings for determining MLH1 status using QuPath.

QuPath Positive Cell Detection	
**Setup Parameters**	
Detection image	Haematoxylin Optical Density
Requested pixel size	0.5 μm
**Nucleus Parameters**	
Background radius	8 μm
Median filter radius	0 μm
Sigma	1.5 μm
Minimum area	10 μm^2^
Maximum area	400 μm^2^
**Intensity Parameters**	
Threshold	0.1
Maximum background intensity	2
Split by shape	Selected
Exclude DAB (membrane staining)	Not selected
**Cell Parameters**	
Cell expansion	5 μm
Include cell nucleus	Selected
**General Parameters**	
Smooth boundaries	Selected
Make measurements	Selected
**Intensity Threshold Parameters**	
Score compartment	Nucleus: DAB Optical Density mean
Threshold 1+	0.2
Single Threshold	Selected

## Data Availability

Data are available upon reasonable request to the corresponding authors.

## Supplementary Materials

The following supporting information can be downloaded at https://www.mdpi.com/article/10.3390/diagnostics13111890/s1, Table S1: Characterisation of the inflammatory bowel disease-associated colorectal cancer tissue microarray; Table S2: Cores flagged by our algorithm for manual review due to equivocal tissue histology.
