# Supplementary material for: QuPath Algorithm Accurately Identifies MLH1-Deficient Inflammatory Bowel Disease-Associated Colorectal Cancers in a Tissue Microarray
Source: Diagnostics (Basel). 2023 May 28;13(11):1890. doi: 10.3390/diagnostics13111890 (PMC10253133; doi:10.3390/diagnostics13111890)
Supplement: Supplementary file 1 [file diagnostics-13-01890-s001.zip › diagnostics-2382482-supplementary.pdf]

Supplementary Materials

Table S1. Characterisation of the inflammatory bowel disease-associated colorectal cancer tissue microarray

Table S2. Cores flagged by our algorithm for manual review due to equivocal tissue histology.

Table S1. Characterisation of the inflammatory bowel disease-associated colorectal cancer tissue microarray

Case	Sex	IBD	Age at IBD-CRC Diagnosis	Site of IBD-CRC	IBD-CRC Histology	Dukes'/ TNM stage
1	F	CD	70	Caecum	Adenocarcinoma	A / pT2N0
2	F	UC	71	Rectum	Adenocarcinoma	B / pT3N0
3	F	CD	85	Caecum	Adenocarcinoma	B / pT3N0
4	M	UC	60	Sigmoid	Adenocarcinoma	C1 / pT4N1
5	F	CD	50	Caecum	Adenocarcinoma	B / pT3N0
6	M	UC	76	Rectum	Adenocarcinoma	C / pT3N1
7	M	CD	79	Right Colon	Signet Cell Adenocarcinoma	C1 / pT4N1
8	F	CD	70	Caecum	Adenocarcinoma	B / pT4N0
9	F	UC	64	Ascending	Adenocarcinoma (<50% mucinous)	B / pT4N0
10	M	CD	35	Ileo-caecal valve	Mucinous Adenocarcinoma	C1 / pT4bN2
11	F	CD	85	Recto-sigmoid	Adenocarcinoma	C1 / pT4N1
12	M	CD	74	Rectum	Adenocarcinoma	B / pT3N0
13	M	CD	65	Caecum	Adenocarcinoma	B / pT4N0
14	M	CD	65	Caecum	Squamous Cell Carcinoma	B / pT4N0
15	M	CD	44	Transverse	Adenocarcinoma	B / pT3N0
16	M	UC	77	Recto-sigmoid	Mucinous Adenocarcinoma	B / pT3N0
17	M	CD	51	Ascending	Adenocarcinoma (<50% mucinous)	D / pT4N1M1
18	M	UC	64	Caecum	Adenocarcinoma	C1 / pT3N1Mx
19	F	CD	69	Ascending	Adenocarcinoma	B / pT3N0
20	F	CD	26	Ano-rectum	Squamous Cell Carcinoma	*pT3N0
21	M	UC	67	Rectum	Adenocarcinoma	A / pT1
22	F	UC	53	Rectum	Mucinous Adenocarcinoma	C1 / pT3N1
23	M	UC	68	Rectum	Adenocarcinoma	A / pT1Nx
24	F	UC	91	Ascending	Adenocarcinoma	B / pT3N0
25	F	CD	64	Hepatic Flexure	Mucinous Adenocarcinoma	B / pT3N0
26	M	UC	46	Splenic Flexure	Adenocarcinoma	C1 / pT3N1
27	M	UC	61	Sigmoid	Mucinous Adenocarcinoma	A / pT1N0
28	M	UC	51	Transverse	Adenocarcinoma	C2 / pT4N2
29	M	UC	85	Recto-sigmoid	Adenocarcinoma	C1 / pT3N1Mx
30	F	UC	33	Ascending	Adenocarcinoma	B / pT3N0
31	F	UC	33	Transverse	Adenocarcinoma	B / pT4N0
32	F	UC	33	Descending	Mucinous Adenocarcinoma	B / pT3N0
33	M	CD	66	Caecum	Adenocarcinoma	B / pT3N0
34	M	UC	80	Rectum	Mucinous Adenocarcinoma	B pT3N0

Cases 13 and 14 represent 2 synchronous IBD-CRC from the same patient. Cases 30, 31 and 32 represent 3 synchronous IBD-CRC from the same patient. *No Dukes' staging applied to ano-rectal squamous cell carcinomas. Abbreviations: M – male; F – female; IBD-CRC – inflammatory bowel disease-associated colorectal cancer; CD – Crohn's disease; UC – ulcerative colitis

Table S2. Cores flagged by our algorithm for manual review due to equivocal tissue histology.

Core number corresponds to our anonymised process for matching cases and not necessarily to specific patients or cores listed elsewhere in manuscript.

Core	Qualitative Review – why the core was flagged for review
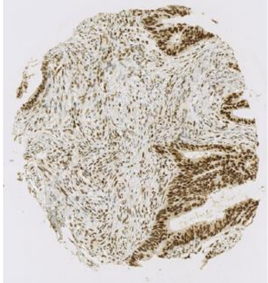 CORE 3 (TUMOUR)	This core has a small quantity of tissue and many tumour cells were misclassified as normal cells (~53.9%).
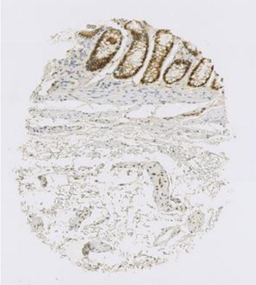 CORE 4 (NORMAL)	This core contains a small quantity of epithelium and many normal cells were misclassified as tumour (~64.8%).
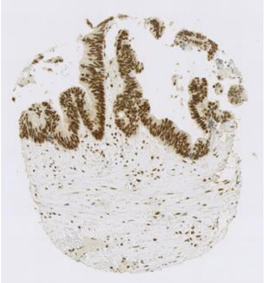 CORE 6 (TUMOUR)	~30.5% of tumour was misclassified as normal epithelium.
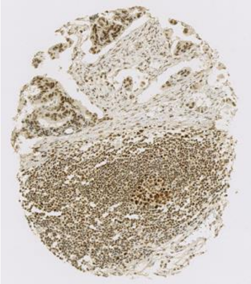 CORE 26 (TUMOUR)	This core has a large immune cell infiltrate and ~31.9% of cells were classified as normal epithelium instead of lymphocytes. Tumour was not misclassified as normal epithelium. There is also only a small quantity of scorable tumour tissue in this core.

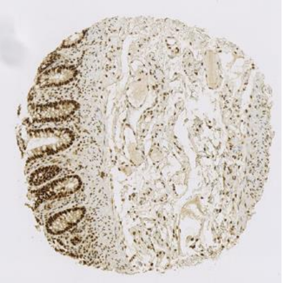 CORE 37 (NORMAL)	This core contains a small quantity of epithelium and many normal cells were misclassified as tumour (~57.1%).
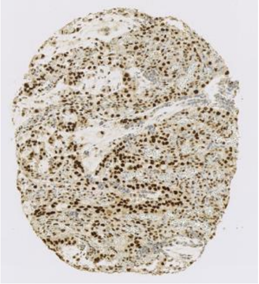 CORE 39 (TUMOUR)	There is atypical tumour morphology. ~72.4% of tumour cells were correctly identified, which just falls short of the pre-determined 75% cut-off.
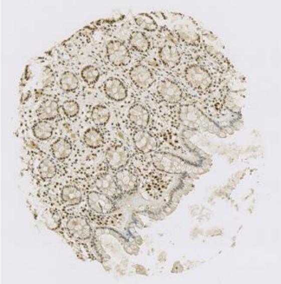 CORE 40 (NORMAL)	This core contains normal mucosal crypts with small nuclei that were misclassified mostly as stroma.
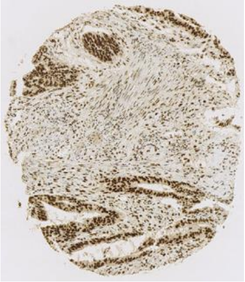 CORE 41 (TUMOUR)	This core has a reasonably small quantity of histologically diagnostic tissue and ~67.6% of tumour cells were misclassified as normal epithelium.
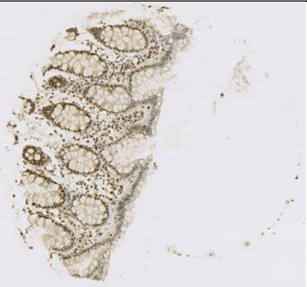 CORE 43 (NORMAL)	This core contains normal mucosal crypts and both normal epithelium (~36.8% correctly) and tumour (~63.2% incorrectly) were identified.

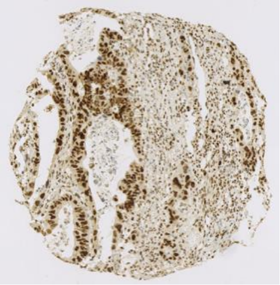 CORE 49 (TUMOUR)	There is atypical tumour morphology. ~74.3% of tumour cells were correctly identified whereas ~25.7% were misidentified as normal epithelium, which just falls short of the pre-determined 75% cut-off. Most errors were made misclassifying larger stromal cells as normal epithelium.
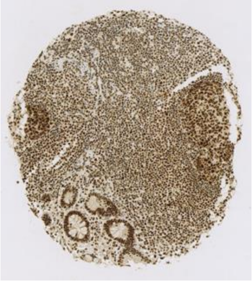 CORE 78 (NORMAL)	This core has a large immune cell infiltrate - lymphoid follicles (3 o'clock and 9 o'clock positions) were misclassified as tumour. Immune cell infiltrate was also misclassified as normal epithelium. There is also only a small quantity of normal epithelium in this core.
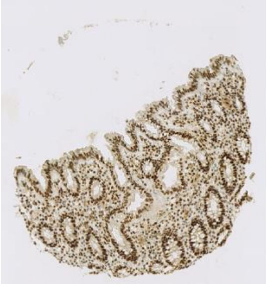 CORE 79 (NORMAL)	This core contains normal mucosal crypts and some normal epithelium and many stromal immune cells were misclassified as tumour.
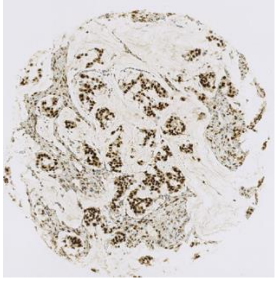 CORE 81 (TUMOUR)	There is atypical tumour morphology (mucinous cell). ~47.1% of tumour cells were misclassified as normal epithelium.
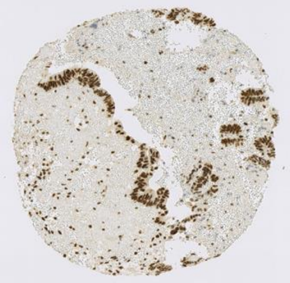 CORE 83 (TUMOUR)	There are a small number of cells with lack of supporting tissue architecture and ~50% of tumour cells were misclassified as normal epithelium.

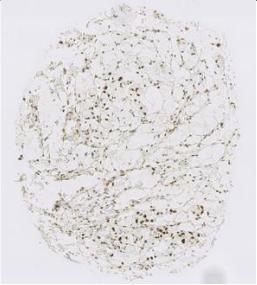 CORE 94 (TUMOUR)	There is atypical tumour morphology (signet ring cell). Most cells were classified as stroma.
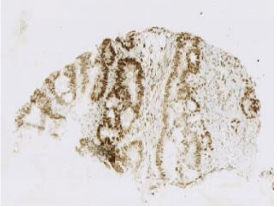 CORE 101 (TUMOUR)	This core has been minorly distorted during either the TMA construction process or microtomy. Some tumour cells were misclassified as normal epithelium or stroma; some tumour cells were not detected.
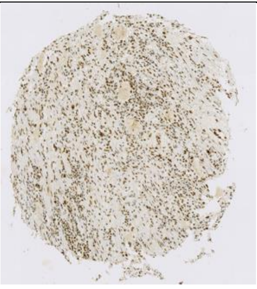 CORE 111 – TUMOUR	This core contains a small quantity of tumour and atypical morphology. Many tumour cells were misclassified as stroma.

Cores flagged by our algorithm for manual review due to equivocal MLH1 status

Core number corresponds to data from Supplementary Data 1 for reference, and not to specific patients listed in Table 1 of the manuscript.

Core	Qualitative Review – why the core was flagged for review
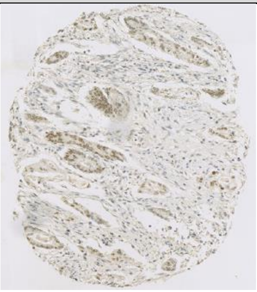 CORE 7 (PROFICIENT)	Immunostain intensity is weak/patchy. Much of the stroma is also MLH1 negative which could infer ineffective epitope retrieval or variation in epitope fixation.
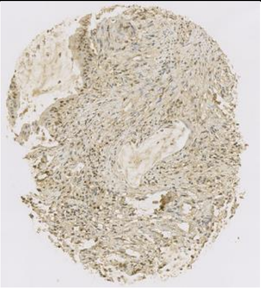 CORE 21 (PROFICIENT)	Immunostain intensity is weak/patchy and there is background staining.

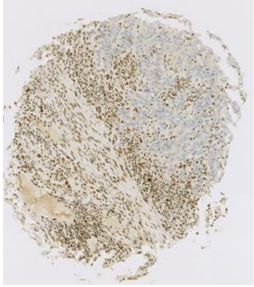 CORE 33 (DEFICIENT)	This core contains a dense MLH1 positive lymphocytic infiltrate and some of these cells were misclassified as normal epithelium or tumour, confounding results.
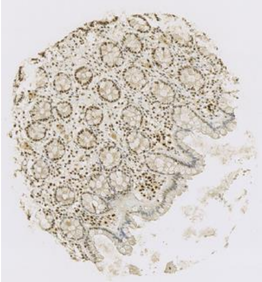 CORE 40 (PROFICIENT)	Many cells in this core were misclassified as stroma (as discussed in Table 1). Therefore, the percentage of cells used to analyse epithelial/tumour expression of MLH1 was significantly reduced. Further, there is a known MLH1 expression gradient, with strong expression observed at the crypt base and weak/absent expression observed at the luminal surface.
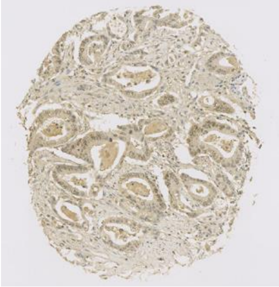 CORE 45 (PROFICIENT)	Immunostain intensity is weak/patchy. Further, there is noticeable non-specific background staining.
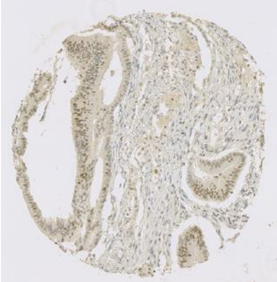 CORE 84 (PROFICIENT)	Immunostain intensity is weak/patchy with evidence of some non-specific background staining.
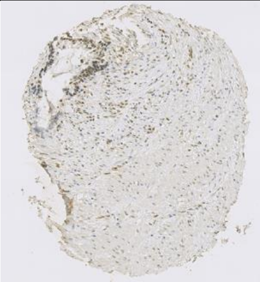 CORE 87 (PROFICIENT)	There is a small quantity of tumour in this core and the Immunostain intensity is weak/patchy.

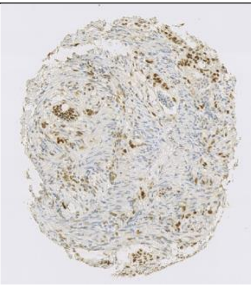 CORE 91 (PROFICIENT)	Areas of stroma were misclassified by our algorithm as tumour, which increased the perceived proportion of ‘negative MLH1’ cells.
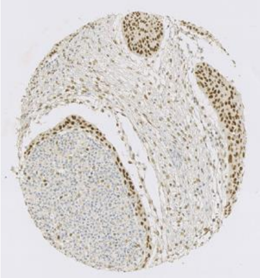 CORE 105 (PROFICIENT)	This core has an MLH1 negative immune cell infiltrate that was misclassified as tumour which confounded output for MLH1 status.
